# Molecular mechanisms of thioridazine resistance in *Staphylococcus aureus*

**DOI:** 10.1371/journal.pone.0201767

**Published:** 2018-08-08

**Authors:** Claes Søndergaard Wassmann, Lars Christian Lund, Mette Thorsing, Sabrina Prehn Lauritzen, Hans Jørn Kolmos, Birgitte Haahr Kallipolitis, Janne Kudsk Klitgaard

**Affiliations:** 1 Department of Biochemistry and Molecular Biology, University of Southern Denmark, Odense, Denmark; 2 Institute of Clinical Research, Research Unit of Clinical Microbiology, University of Southern Denmark, Odense, Denmark; University of Padova, Medical School, ITALY

## Abstract

*Staphylococcus aureus* has developed resistance towards the most commonly used anti-staphylococcal antibiotics. Therefore, there is an urgent need to find new treatment opportunities. A new approach relies on the use of helper compounds, which are able to potentiate the effect of antibiotics. A well-studied helper compound is thioridazine, which potentiates the effect of the β-lactam antibiotic dicloxacillin against Methicillin-resistant *Staphylococcus aureus* (MRSA). In order to identify thioridazine’s mechanism of action and how it potentiates the effect of dicloxacillin, we generated thioridazine resistant strains of MRSA USA300 by serial passage experiments. Selected strains were whole-genome sequenced to find mutations causing thioridazine resistance. Genes observed to be mutated were attempted deleted in MRSA USA300. The *cls* gene encoding a cardiolipin synthase important for synthesis of the membrane lipid cardiolipin was found to be mutated in thioridazine resistant strains. Deletion of this gene resulted in a two-fold increased Minimum inhibitory concentrations (MIC) value for thioridazine compared to the wild type and decreased susceptibility similar to the thioridazine resistant strains. Since cardiolipin likely plays a role in resistance towards thioridazine, it might also be important for the mechanism of action behind the potentiating effect of thioridazine. TDZ is known to intercalate into the membrane and we show here that TDZ can depolarize the plasma membrane. However, our results indicate that the membrane potential reducing effect of TDZ is independent of the resistance mechanism.

## Introduction

Methicillin-resistant *Staphylococcus aureus* (MRSA) is a globally growing health-care problem. Patients suffering from infections caused by MRSA have a higher mortality [1] and are hospitalized longer [2] compared to infections caused by methicillin-susceptible *S*. *aureus* (MSSA). This is partly due to a lack of effective, well-proven antibiotics besides vancomycin. Resistance in MRSA constitutes a global health problem, while the discovery and development of novel antibiotics is stagnating.

In recent years, treatment of infections caused by multi-drug resistant bacteria with antibiotic/adjuvant combinations has become a possibility [3]. Thioridazine (TDZ) was originally developed as an anti-psychotic drug and a member of the phenothiazine family, which mostly features various neuroleptic and anti-histaminergic drugs. It was brought to market as "Mellaril" and approved for treatment of schizophrenia and psychosis; however it was globally withdrawn in 2005 because of its tendency to cause severe side effects when used regularly. TDZ has been shown to have a synergistic effect with a β-lactam antibiotic at low concentrations *in vitro* by restoring susceptibility to β-lactam antibiotics in otherwise resistant organisms [4, 5].

Thorough evidence of the bactericidal activity of TDZ has been collected, both *in vitro* and *in vivo* [4–12], but the mechanism of action (MOA) remains unknown. The question on how TDZ and the combination of TDZ and a β-lactam antibiotic reduce the survival of bacterial growth has not been fully answered yet. Studies on synthetic membranes have shown that TDZ intercalates in the lipid bilayer through interactions with negatively charged phospholipids at the polar/apolar interface of the bilayer, disturbing various membrane associated processes [13]. Whether TDZ increases or decreases the fluidity of membranes is not clear since studies have reported both outcomes [13, 14]. The precise target of TDZ in the membrane, and how it affects bacteria like *S*. *aureus*, still remains unclear.

The synergistic effect of TDZ in combination with antibiotics has been proven to be independent of resistance to DCX [5]. The expression of cell wall and stress response related genes is affected upon TDZ exposure, including *vraSR* and the genes regulated by this two-component system [6]. *vraSR* is induced through cell wall damage, indicating that TDZ alone affects the integrity of the cell wall [6]. Global transcriptional analyses have shown that TDZ exposure induces genes involved in early stage peptidoglycan biosynthesis [7]. In this study, TDZ was additionally shown to interfere with the formation of the pentaglycine bridge of peptidoglycan precursors in the cell wall biosynthesis pathway. This indicates that TDZ has pleiotropic effects on factors associated with the cytoplasmic membrane and/or cell wall—but the target molecule remains unknown.

An *in vivo* study showed that the combination treatment can clear the bacterial load from the intestine of the nematode model organism, *Caenorhabditis elegans*, compared to monotherapy [8]. Furthermore, recent data showed a reduction of an intraperitoneal infection by intraperitoneal treatment in mice [9], but not in a porcine aorta graft model experiment, which revealed no statistical differences for bacteriological endpoints between the treatment groups [12]. However, in the same porcine model, drug-loaded Interpenetrating polymer networks (IPNs) catheters were found to significantly decrease the frequency of central venous catheter-related infections [10].

The aim of the present study was to identify the mechanism of action for thioridazine and its ability to potentiate the effect of dicloxacillin. We investigated this by genotypic and phenotypic analysis of induced TDZ resistance in MRSA USA300 through a serial passage experiment. Our results suggest that TDZ resistance has similarities to daptomycin resistance and that cardiolipin takes part in the mechanism of TDZ resistance.

## Methods and materials

### Strains and growth conditions

The bacterial strain used in this study is the parental *S*. *aureus* MRSA strain USA300 FPR3757 (ATCC BAA-1556) [15]. Growth media used are Müller Hinton broth (MH) (Merck) and Brain Heart Infusion broth (BHI) (Oxoid). All strains were grown at 37°C as standard. For cloning, *E*. *coli* strain IM08B was grown in Lysogeny broth (LB) (Sigma Aldrich). Antibiotics (chloramphenicol (CHL) and anhydrotetracycline (ATc)) were added when required. See S1 Table for an overview of all strains in the present study.

### *In vitro* selection for increased thioridazine tolerance

The MRSA strain USA300 was passed through multiple cultures with progressively higher concentrations of TDZ. Following overnight growth, the culture grown at the highest concentration of TDZ was diluted 1:1000 in fresh medium containing an equal or higher (≤ 10 μg/ml increments) TDZ concentration and the subsequent culture was allowed to incubate at 37 °C with shaking. Starting with 32 μg/ml TDZ at day 0, the cycle was repeated every day reaching 90 μg/ml TDZ at day 38.

### Growth assays

Using an oCelloScope (Philips BioCell A/S), the bacterial density was measured over a period of 24 hours. The background corrected absorption (BCA) was measured, which roughly translates to conventional measurements of turbidity. A 96-well plate (Nunc Edge) was prepared with different concentrations of TDZ and DCX in MH media. Diluted overnight cultures (OD_600_nm of 0.005) were added to each well. The plate was incubated at 37 °C (without shaking) for 24 hours. For comparison, the same concentrations of TDZ and DCX were used for all strains which were 16 μg/ml for TDZ and 0.125 μg/ml for DCX which as previously published shows synergy [5]. Experiments were repeated with at least three biological replicates.

### Minimum inhibitory concentrations

The MIC was determined using the broth microdilution method [16]. The MIC was interpreted as the lowest concentration at which no bacterial growth was observed. Values given are the consensus MIC derived from at least two independent determinations. Briefly, MIC measurements were performed using the MH medium using 96-well plates (Nunc A/S). A total volume of 100 μL with a bacterial inoculum of 5×10^5^ cfu/mL was incubated with two-fold dilution series of DCX and/or TDZ at 37°C for 18–20 hours with shaking. Bacterial growth was determined by turbidity measurements. Experiments were repeated with at least three biological replicates.

### Purification of genomic DNA

Genomic DNA was purified using the GenElute Bacterial Genomic DNA Kit (Sigma-Aldrich) according to the manufacturer’s recommendations. The enzymatic lysis solution was prepared with lysozyme and lysostaphin (Sigma-Aldrich) as recommended by the manufacturer.

### DNA sequencing and library preparation

DNA sequencing and library preparation were conducted at the National High-throughput DNA sequencing Centre at the University of Copenhagen. The complete sequencing data set is available at the NCBI BioProject database under accession number PRJNA449536 and the NCBI Sequence Read Archive (SRA) database under accession number SRP139353. Library preparation was done according to the protocol specified by Meyer and Kircher [17] with a target insert size of 600 nt. Sequencing was carried out on an Illumina MiSeq benchtop sequencer as paired end sequencing with a read length of 250 base pair (bp).

### Preprocessing of reads

Reads were obtained in .fastq format. Sequencing adapters were removed and resulting reads trimmed according to phred score. Reads below 36 bp in length were discarded.

### Identification of variants

Single-nucleotide polymorphisms (SNPs) were identified using three different workflows, two based on reference sequence alignment and one based on *de novo* assembly. For sequence alignment, reads were aligned against the reference genome of *S*. *aureus* USA300 FPR3757. For genome alignment both “Stampy”[18] and “Breseq” [19] were used. For Stampy alignments, optical duplicates were removed using “Picard Tools” (http://broadinstitute.github.io/picard). The “GATK” was used to improve alignment around indels. Variants were called using GATKs UnifiedGenotyper (UG). For breseq variants, only variants classified as “strong evidence” were considered. *De novo* assembly and variant calling based hereupon were conducted using “cortex_var”[20], using the “independent” workflow. Variants identified by UG and cortex were filtered according to quality scores. All variants were annotated using “SnpEff”[21] and the impact on protein level estimated using the “Project HOPE”[22] webserver. Variants present in the wild type strain or in only a single mutant strain were discarded.

### Phylogenetic analysis

“Wombac” (http://www.vicbioinformatics.com/software.wombac.shtml), using default parameters, was used to generate a phylogenetic tree of all sequenced strains.

### Creation of deletion mutants

Plasmids for in-frame deletions of relevant genes (*rpoC*, *SAUSA300_0649*, *SAUSA300_0703*, *SAUSA300_0911*, *pyc*, *SAUSA300_1119*, *SAUSA300_1720*, *SAUSA300_1797*, and *cls*) were constructed through splicing by overlap extension (SOE) PCR [23] into the pIMAY plasmid [24]. The specific primers used for creation of deletion mutants can be found in S2 Table. Briefly, the plasmids containing a sequence up- and downstream of the gene of interest were transformed *into Escherichia coli* IM08B, purified, and transformed into electrocompetent *S*. *aureus* (USA300). Positive colonies grown on BHI plates containing 20 μg/ml CHL were identified by colony PCR using primers IM151 and IM152 [24]. Genomic integration was performed in BHI broth with 20 μg/ml CHL at 28 °C overnight (ON). Colony PCR, using the primer pairs outF & D and A and outR, was conducted to determine, if the knockout construct was inserted up- or downstream of the gene of interest. Excision of pIMAY plasmid was performed by repeated overnight culture dilutions in BHI at 28 °C for a minimum of 7 days. The culture was plated on BHI agar plates with and without 1 μg/ml anhydrotetracycline and incubated at 28 °C for 2 days. Colonies were subsequent spotted on BHI plates with and without 20 μg/ml CHL. Verification of plasmid excision was performed by colony PCR with outF and outR primers. The deletion of the gene was verified by sequencing by Eurofins Genomics for the genes *SAUSA300_0649*, *SAUSA300_0911*, *pyc*, *SAUSA300_1797*, and *cls*. The remaining deletion mutants were unsuccessful for different technical reasons.

### Assessment of membrane potential using flow cytometry

Measurement of the membrane potential was conducted using the BacLight Bacterial Membrane Potential Kit (Invitrogen) in three biological replicates. An overnight culture was diluted to an OD_600_nm of 0.02 in BHI and incubated at 37 °C until early exponential phase (OD_600_nm of 0.3). The culture was then diluted 1:100 in sterile phosphate-buffered saline. Four dilutions were made per sample. One sample was treated with 5 μM of CCCP (carbonyl cyanide 3-chlorophenylhydrazone) to depolarize the cells, thereby acting as a depolarized control and another sample was treated with 16 μg/ml of TDZ and left at room temperature for 5 minutes. The depolarized control, the TDZ treated sample, and one untreated sample were stained for 15–30 minutes with 30 μM of DiOC_2_ dye and the last sample was left as an unstained control. After staining, the samples were analyzed in a BD FACS Aria II flow cytometer (Becton, Dickinson and Company). 10^4^ events were analyzed, while exciting the particles with a laser emitting 488 nm and collecting the green and red fluorescence. As a measure of the membrane potential, the ratio of the mean red fluorescence intensity compared to the mean green fluorescence intensity was calculated. Cells with unaffected membranes will have an accumulation of the dye which causes a red fluorescence emission and thus a high red/green ratio whereas cells with affected membranes will have low dye content causing green fluorescence emission and thus a low red/green ratio.

## Results

### Generation and sensitivity of the TDZ resistant strains

A serial passage with increasing concentrations of TDZ was conducted to evaluate the potential of MRSA to acquire resistance towards TDZ (see S1 Fig). The experiment resulted in adaptation to TDZ with a two-fold increase in MIC over a period of 39 days.

**Fig 1:**
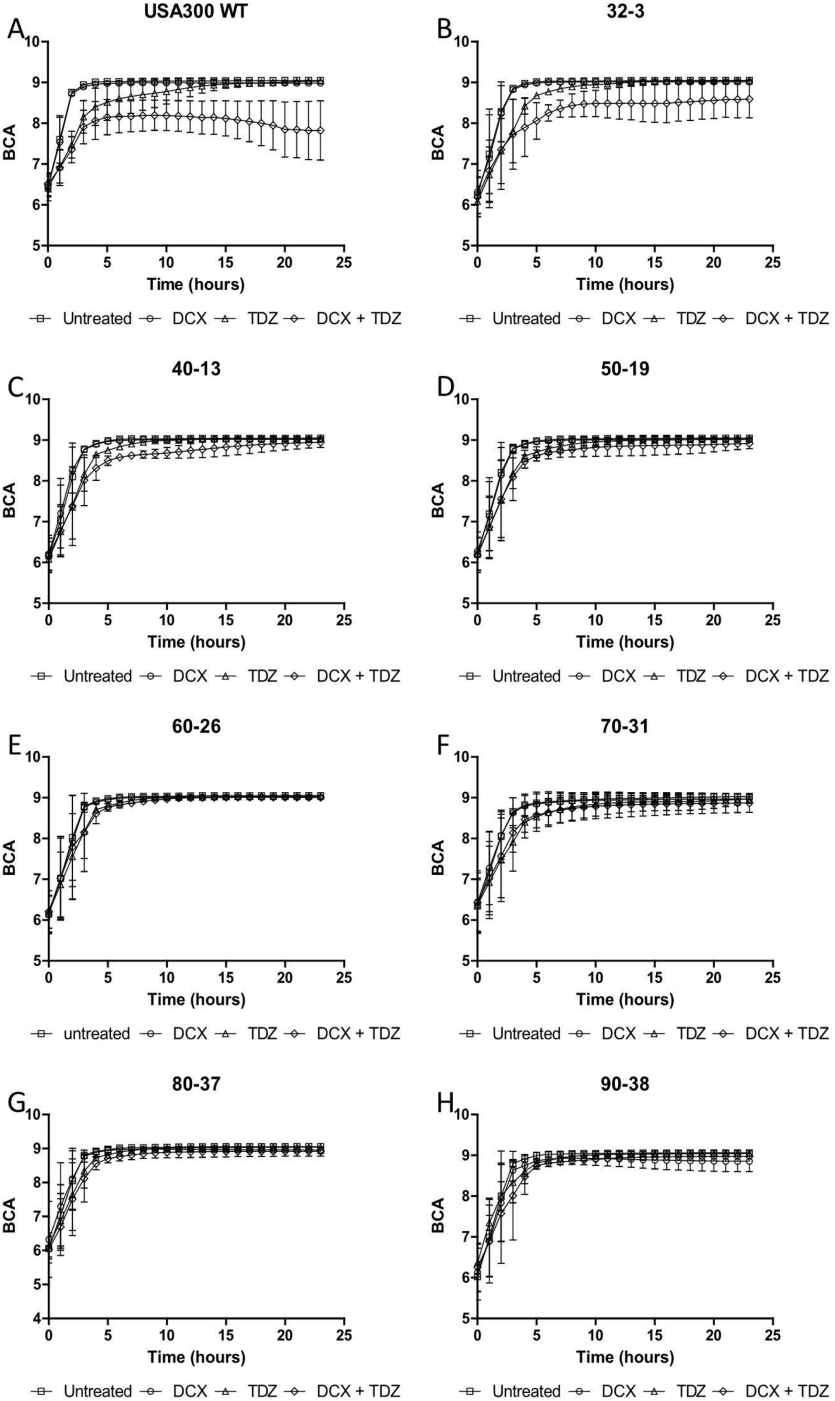
Growth assays. Effect of TDZ (16 μg/ml) and DCX (0.125 μg/ml) on growth and growth inhibition of USA300 wild type and derivative strains 32–3, 40–13, 50–19, 60–26, 70–31, 80–37, and 90–38 obtained from serial passage experiment with TDZ.

Growth assays on TDZ resistant strains isolated were conducted at different time points during the serial passage. The growth of the strains was monitored during exposure to the combined treatment of TDZ and DCX and compared to the growth when exposed to the individual compounds. Fig 1 shows that the synergistic effect between TDZ and DCX disappears as the bacteria acquire resistance towards TDZ. Wild type, strain 32–3, and strain 40–13 remain susceptible to the combination of TDZ and DCX. There is a significant inhibition of bacterial growth for these strains, when comparing the combined treatment with monotherapy (Fig 1a–1c). Strains 50–19, 60–26, 70–31, 80–37, and 90–38 exhibit no difference in inhibition of growth when comparing combined treatment and monotherapy (Fig 1d–1h). From this, it can be concluded that synergy is lost at the TDZ concentration which previously gave synergy in combination with the β-lactam antibiotic DCX. The emergence of resistance in strain 50–19 and later strains indicates key mutational events having taken place in the serial passage after the culture was exposed to 50 μg/ml of TDZ. On the basis of this finding, strain 50–19 and later strains were chosen for whole-genome sequencing.

### Whole-genome sequencing of TDZ resistant strains

For detection of genotypic changes, whole-genome sequencing was conducted on five TDZ resistant strains and the wild type, *S*. *aureus* USA300 FPR3757. Sequence data were analyzed in regards to SNPs, insertions, and deletions. Variants were called with three different workflows. Comparison of the whole-genome sequences of our isolate of USA300 FPR3757 to the online genomic sequence revealed SNPs, which were excluded before further analyses of genomic variations to the TDZ resistant strains. Overall 10 different SNPs and one 13 bp insertion were detected. Nine of the SNPs were detected through the Stampy, Breseq and cortex var variant calling. A tenth SNP was only detected through the Stampy and Breseq workflow. The 13 bp insertion was detected through cortex var and Breseq variant calling, while the combination of Stampy and UnifiedGenotyper did not detect the mutation. Three variants were not included for further analysis, since only one workflow identified these (1 Stampy, 2 cortex var). Of the 10 final SNPs, one introduced a premature stop codon, whilst the others were missense mutations. Details about filtering of the mutations can be seen in supplementary S2 and S3 Figs. All mutations were confirmed through Sanger sequencing. Table 1 show the mutations identified through whole-genome sequencing with indications of the affected genes, their functions, as well as the probable mutational consequences as identified by the Project HOPE web server. Biologically, the affected genes can be categorized into three groups: Cell wall related (*SAUSA300_0703*, *SAUSA300_1720*, and *cls*), cytoplasmic membrane related (*SAUSA300_0649*, *SAUSA300_0911*, and *SAUSA300_1720*), and others (*rpoC*, *pyc*, *SAUSA300_1119*, and *SAUSA300_1797*).

**Table 1:** Mutations identified through whole-genome sequencing of TDZ resistant mutants.

Affected gene	Gene function	Position	Strains	Codon change	AA change	Effect	Domain	Probable mutational consequences
***rpoC***	RNA polymerase	590016	50–19 60–26	Cgt = > Tgt	R = > C	Missense	N-terminal	Lost/affected nucleic acid binding
***SAUSA300_0649***	Phosphate transport^a^	724529	50–19 60–26 70–31 80–37 90–38	aTg = > aAg	M = > K	Missense	Putative phosphate transport regulator	Lost/affected multimeric interactions
***SAUSA300_0703***	Lipoteichoic acid synthetase^a^	778099	70–31 80–37 90–38	Gcc = > Ccc	A = > P	Missense	Sulfatase, Alkaline-phospha-tase-like, core domain	Disturbance of main activity
***SAUSA300_0911***	Na+/H+-antiporter^a^	999109	70–31	gCc = > gAc	A = > D	Missense	Substrate-specific transporter activity	Affect hydrophobic interactions within the protein or with membrane lipids
***SAUSA300_0911***	Na+/H+-antiporter^a^	999438	50–19 60–26	Cga = > Tga		Stop gained		Truncated protein, loss of function
***pyc***	Pyruvate carboxylase	1109416	70–31 80–37 90–38	aAa = > aTa	K = > I	Missense	ATP-binding domain	Structural disturbance of domain and loss of function
***SAUSA300_1119***	Dihydroxy-acetone kinase^a^	1224681	80–37 90–38	Gga = > Aga	G = > R	Missense	DAK2 domain	Disturbance of conformation
***SAUSA300_1720***	Peptido-glycan cleavage^a^	1903228	70–31 80–37 90–38	Cat = > Tat	H = > Y	Missense	Amidase domain, hydrolase activity	Disturbance of folding, loss of function
***SAUSA300_1797***	Transcrip-tional regulator^a^	1980241	50–19 60–26	Gaa = > Caa	E = > Q	Missense		Disturbance of function
***cls***	PG to CL conversion	2208587	50–16 60–26			13BP insertion		Frame shift. Loss of function
***cls***	PG to CL conversion	2209475	70–31 80–37 90–38	gCt = > gAt	A = > D	Missense	Cardiolipin synthase, Phospho-lipase D-like domain	Disturbance of function

^a^ hypothetical protein, function inferred from homologous proteins.

The five TDZ resistant strains were additionally inspected for large insertions and deletions. No areas with zero coverage longer than 500 bp were detected in the reads of all strains. Therefore, it is safe to assume that no major deletions occurred in the mutants’ genome under the serial passage experiment. *De novo* assembly of unmapped reads resulted in 9 to 14 contigs, depending on the sample, greater than 1000 bp in length per strain. All contigs mapped to *S*. *aureus* USA300 FPR3757’s plasmid sequences pUSA01, pUSA02 and pUSA03, except one 5386 bp contig, representing a bacteriophage added during sample preparation, PhiX 174 [25]. Therefore, no large insertions were found in the analysis.

### Phylogenetic analysis of the TDZ mutants

**Fig 2:**
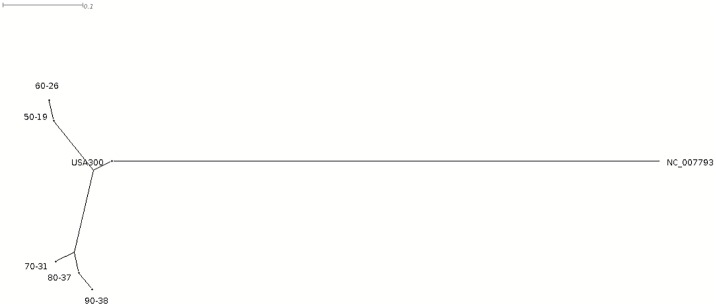
Phylogenetic analysis. Comparative phylogenetic tree analysis shows USA300 wild type and TDZ resistant strains based on genome analysis together with the genomic reference sequence for *S*. *aureus* USA300_FPR3757 (NC_007793).

From the sequencing data, a phylogenetic tree was made (Fig 2). The tree shows two lineages of bacteria which evolved separately under the serial passage. This coincides with the two distinct groups of mutations listed in Table 1. Strains 50–19 and 60–26 share mutations in the genes *rpoC*, *SAUSA300_0911*, *SAUSA300_1797* and strains 70–31, 80–37 and 90–38 share mutations in the genes *SAUSA300_0649*, *SAUSA300_0703*, *pyc*, *SAUSA300_1119*, *SAUSA300_1720*, and *cls*. Strains 70–31, 80–37, and 90–38 are the most resistant strains, therefore mutations in *SAUSA300_0649*, *SAUSA300_0703*, *pyc*, *SAUSA300_1119*, *SAUSA300_1720*, and *cls* may be of greater interest in regards to TDZ resistance.

### Analysis of mutated genes

To clarify the importance of every mutated gene in the TDZ adaptation, single knockout mutants of the genes with mutations identified through whole-genome sequencing were created. We attempted to generate knockout mutants for all 9 genes, but only Δ *SAUSA300_0649*, Δ *SAUSA300_0911*, Δ *pyc*, Δ *SAUSA300_1797*, and Δ *cls* were successfully generated and subjected to further analysis.

For phenotypic analysis of the deletion mutant strains, MICs for TDZ and DCX were determined. With respect to TDZ, the MIC was unchanged for the deletion mutants of USA300_*0911*, *pyc*, USA300_*1797*, and USA300_*0649* at 64 μg/ml when compared to wild type. Strain 80–37 and Δ *cls* exhibited two-fold increased MICs to 128 μg/ml compared to wild type (Table 2). This indicates that a single mutation in the *cls* gene may be sufficient to create resistance towards TDZ. With respect to DCX, the MICs did not differ considerably from wild type in any strain.

**Table 2:** Minimal inhibitory concentrations (MICs) for thioridazine and dicloxacillin. Data given are the mean of at least three replicates ± standard error of the mean (SEM).

	Thioridazine ± SEM (μg/ml)	Dicloxacillin ± SEM (μg/ml)
**WT**	64 **± 16**	0.25 **± 0.06**
**80–37**	128 **± 0**	0.25 **± 0**
**Δ*cls***	128 **± 0**	0.25 **± 0**
**Δ*SAUSA300_0911***	64 **± 30**	0.125 **± 0**
***Δpyc***	64 **± 0**	0.125 **± 0.03**
**Δ*SAUSA300_*1797**	64 **± 0**	0.5 **± 0.11**
**Δ*SAUSA300_*0649**	64 **± 0**	0.125 **± 0**

**Fig 3:**
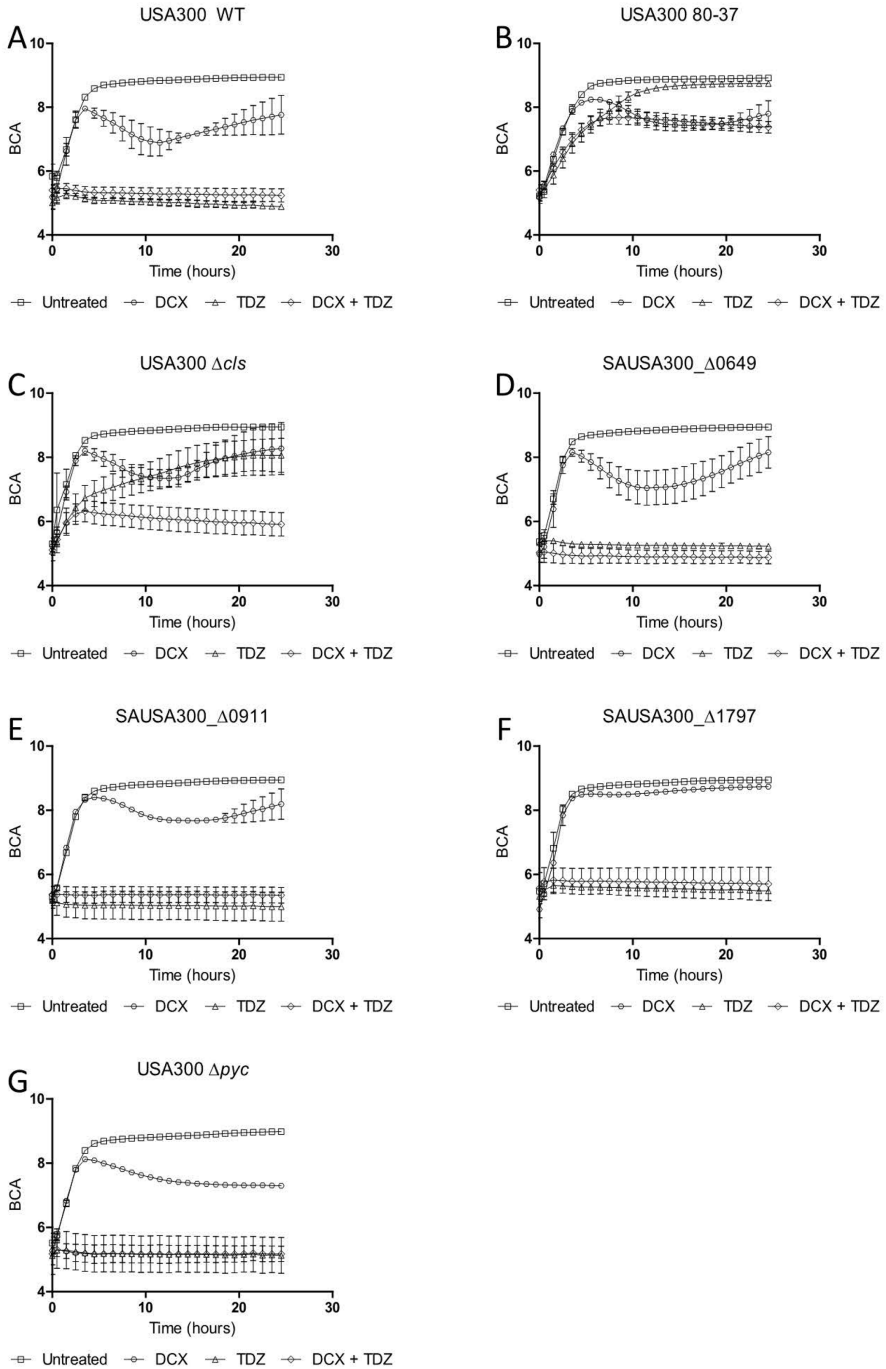
Growth assays. Effects of TDZ (32 μg/ml) and DCX (0.125 μg/ml) on growth and growth inhibition of USA300 wild type, TDZ resistant strain 80–37, Δ *SAUSA300_0649*, Δ *SAUSA300_0911*, Δ *SAUSA300_1797*, and Δ *cls*.

To further analyze the phenotypes of the deletion mutants, growth assays were conducted in biological triplicates. The concentration of TDZ was increased in consequence to the TDZ resistance in the strains. Strains USA300 and 80–37 were used as a negative and positive control, respectively in regards to TDZ resistance and the efficacy of the combination of TDZ and DCX. The deletion mutants Δ*SAUSA300_0649*, Δ *SAUSA300_0911*, Δ *SAUSA300_1797*, and Δ *pyc* exhibited approximately the same phenotype as the wild type, both when treated with TDZ alone and the combined treatment, indicating that these genes do not contribute to the TDZ adaptation (Fig 3). In regards to DCX, the strain Δ *SAUSA300_1797* shows an increased resistance, which may imply a role of the gene in DCX resistance. The deletion mutant Δ *cls* followed the growth curve of 80–37, when exposed to 32 μg/ml of TDZ. When exposed to both TDZ and DCX, the strain exhibits a similar phenotype as the wild type and very different to 80–37. This means that loss of the *cls* gene in *S*. *aureus* leads to a reduced susceptibility towards TDZ, but not the combination of TDZ and DCX. This suggests that cardiolipin affects the bactericidal effect of TDZ and that it is different from the potentiating effect of TDZ. Furthermore, the comparison between Δ *cls* and 80–37 when exposed to TDZ and DCX indicates that other genes than *cls* are responsible for the reduced susceptibility towards the combined treatment as the synergistic effect is present in Δ *cls* and not in 80–37. It is therefore likely that TDZ affects several cellular processes.

### Depolarization of cytoplasmic membrane

**Fig 4:**
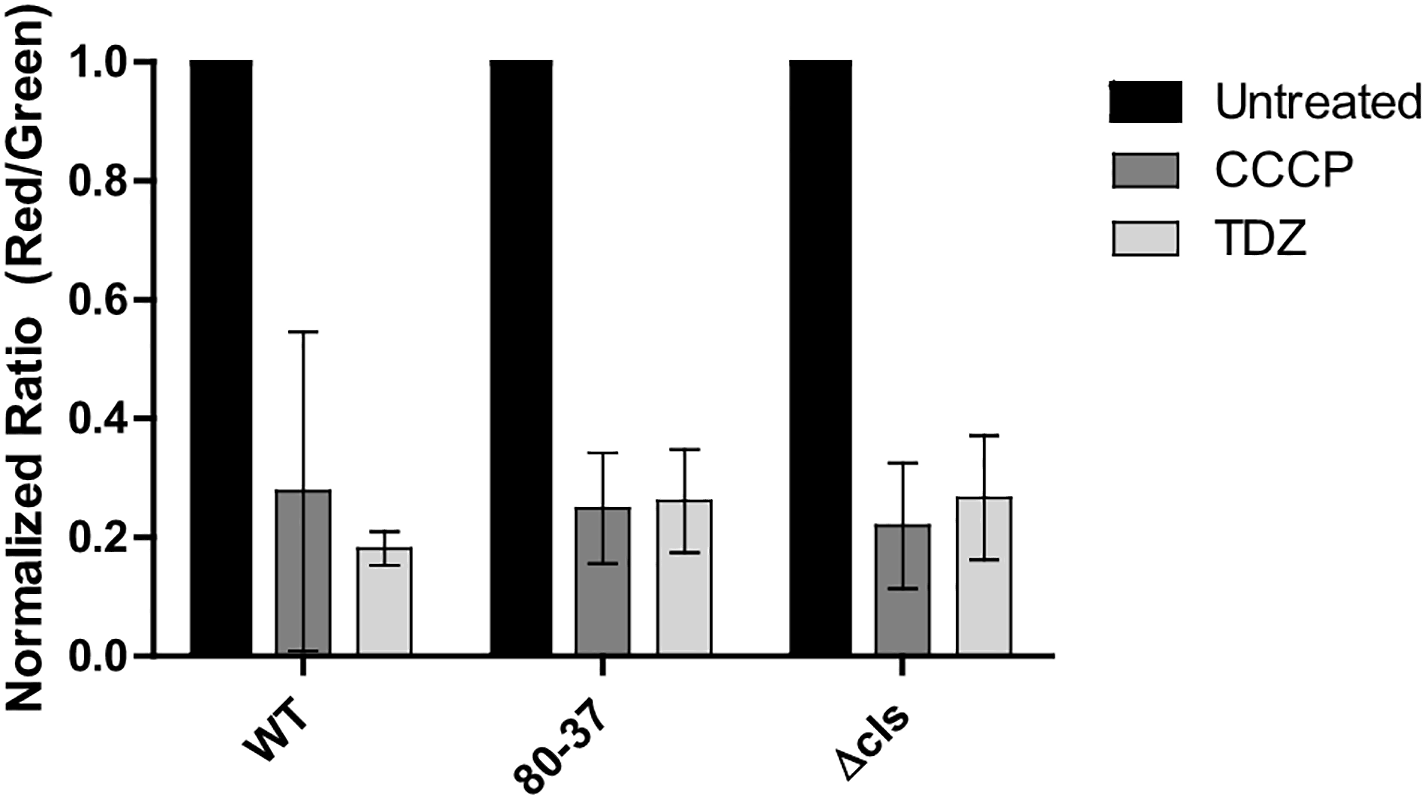
Assessment of membrane potential using flow cytometry. Membrane depolarization assay showing the effect of 16 μg/ml TDZ on the membrane potential of USA300 wild type, TDZ resistant strain (80–37), and Δ *cls*. Bar chart showing red/green mean fluorescence intensity ratio where a high ratio indicates high membrane potential and a low ratio a depolarized membrane. CCCP (2-[2-(3-Chlorophenyl)hydrazinylyidene]propanedinitrile) is a oxidative phosphorylation uncoupler used as a positive control for membrane potential depolarization. Data are normalized against untreated control and show mean values of three biological replicates and standard deviations.

We evaluated TDZ effects on cell membrane depolarization and potential loss in wild type, 80–37, and Δ *SAUSA300_cls* strains under sub-inhibitory concentration of TDZ. The red/green fluorescence ratio for the wild type, 80–37 and Δ *SAUSA300_cls* is shown in Fig 4. CCCP was used as positive control for depolarization of the membrane. The same depolarizing effect can be observed when comparing bacteria treated with TDZ to the untreated controls, showing that TDZ depolarizes the membrane independently of the genetic background of the strains.

## Discussion

### Clinical consequences of TDZ resistance

MRSA can acquire TDZ resistance. This resistance may be a problem for the future clinical use of the combination TDZ and DCX, since the growth assays show that TDZ resistance also leads to a reduced susceptibility towards the combined treatment. Resistance against TDZ manifested as a two-fold increase in MIC from 64 μg/ml to 128 μg/ml after exposure to increasing concentrations of TDZ over a period of 39 days, yet it was not possible to force the bacteria to a higher MIC, as the bacterial growth was inhibited by concentrations above 90 μg/ml. Since the steady state concentration of TDZ in plasma is about 1 μg/ml [26], manifestation of resistance in patients treated with the combined treatment of TDZ and DCX seems unlikely to occur.

The serial passage was conducted in a monoculture *in vitro*. However, the conditions that need to be met for evolution of TDZ resistance could be lower *in vivo*.

### Implication of mutations accumulated under adaptive evolution experiment

All mutations identified through whole-genome sequencing were systematically investigated through review of the existing literature and relevant databases. No overlap between the genes that were mutated through long-time exposure to TDZ and a microarray study of USA300 exposed to TDZ was found [7]. Each identified mutation was therefore evaluated in regards to how they compare to current knowledge of the biological effects and MOA of TDZ.

*rpoC* encodes a DNA directed RNA polymerase subunit. Mutations in the *rpoC* gene have been associated with antibiotic resistance towards cephalosporin [27], daptomycin (DAP) [28, 29] and vancomycin [30, 31] in other organisms. Mutations in this gene have also been associated with optimal growth in minimal media [32], including the MH-medium employed in the adaptive evolution experiment. We did not succeed in producing an *rpoC* deletion mutant. Since RpoC is a component of RNA polymerase, it may not be possible to create a full deletion of the gene. Generation of the missense mutation may be possible to reproduce, even though single nucleotide mutations in *rpoC* have been shown to be unstable [31].

The gene *SAUSA300_0649* encodes a putative phosphate transport regulator in the cytoplasmic membrane. The amino acid sequence of this transporter shows homology to the *phoU* phosphate transport regulator and many other postulated prokaryotic phosphate transport proteins. Very little information is available on this protein. PhoU impacts on widespread processes beyond inorganic phosphate (Pi) transport and acts as a persister switch in *E*. *coli* multidrug tolerance [33]. The mutations were present in all TDZ resistant strains regardless of lineage. However, TDZ MICs were unaffected by the deletion of the gene.

*SAUSA300_0703* or *ltaS* encodes a glycerol phosphate lipoteichoic acid synthase in *S*. *aureus*. Lipoteichoic acid (LTA) is one of the major components of the staphylococcal cell envelope and the enzyme responsible for synthesis of LTA is the membrane embedded LtaS. In *Enterococcus faecium*, a lipoteichoic acid synthesis inhibitor has a bactericidal effect in combination with DAP suggesting that the LtaS enzyme is a suitable target for the development of novel antimicrobial treatment [34]. Multiple studies link defects in the LTA synthesis pathway to hyper-susceptibility towards DAP. DAP interacts with the bacterial cytoplasmic membrane, like TDZ, and aggregates there, ultimately leading to a rapid depolarization of the membrane and inhibition of life sustaining processes in the bacterium [35]. LTA and wall teichoic acids (WTA) play a key role in controlling the net charge of the membrane [36]. TDZ interacts with the negatively charged phospholipids in the cytoplasmic membrane [13]. Such a change in the overall membrane charge and composition is seen in the depolarization of the cytoplasmic membrane by TDZ in this study indicating that an impaired LTS synthesis may explain the phenotypes of the TDZ resistant strain. The predicted effect of the mutation in the *ltaS* gene is to destroy the sulfatase domain of LtaS. It is reasonable to assume that this would disrupt the protein’s function and result in a lower amount of LTA in the cell envelope. LTA is associated with protection against cell wall stressors (DAP, vancomycin and others) [37], therefore it seems unclear how a reduced amount of LTA should confer resistance to TDZ. Unfortunately, we did not succeed in deleting the *ltaS* gene in USA300 so its role in TDZ resistance remains to be clarified.

The gene *SAUSA300_0911* encodes a hypothetical monovalent cation proton antiporter-2 (CPA2) family protein and is assumed to be a sodium hydrogen antiporter. No studies investigating antibiotic resistance and this family of transporter proteins have been conducted so far, making it challenging to estimate the biological relevance of this mutation. Two different mutations were identified through WGS in this gene which was a missense mutation in strain 70–31 and a nonsense mutation in strains 50–19 and 60–26. This hypothetical Na^+^/H^+^ exchanger could be involved in upholding the proton gradient of the cytoplasmic membrane and thereby the membrane potential. The proximity to TDZ’s proposed site of action and it being a nonsense mutation could indicate a role of *SAUSA300_0911* in TDZ resistance mechanism. However, deletion of the *SAUSA300_0911* gene did not restore the sensitivity to TDZ as seen by MIC analysis and growth assay.

A missense mutation in the *pyc* gene encoding a pyruvate carboxylase in *S*. *aureus* was identified in three out of five TDZ resistant mutants. Although pyruvate metabolism is a part of many important processes in the bacterium, no direct interaction with the cytoplasmic membrane, glycine transport or other processes known to be affected by TDZ has been established, and a strain with the *pyc* gene deleted did not show the pyruvate carboxylase to be involved in the TDZ resistance mechanism.

SAUSA300_1119 is a hypothetical protein showing structural similarity to glycerone (dihydroxyacetone) kinases and belongs to a protein family of unknown function that contains an N-terminal DAK2 domain. No knockout mutant was successfully made for this gene and due to a general lack of literature indicating any relevancy for TDZ’s MOA or mechanism of resistance (MOR), the significance of the gene cannot be evaluated at this point.

It can be inferred from the sequence of *SAUSA300_1720*, that the encoded protein is a homolog of an autolysin in *S*. *aureus subsp*. *aureus* MRSA252. *atl* is the major autolysin gene in *S*. *aureus* and has been found to have lowered expression levels in vancomycin-intermediate *Staphylococcus aureus* (VISA) compared to their vancomycin-sensitive *Staphylococcus aureus* (VSSA) parent strains. The reduced *atl* expression levels result in a thickened cell wall [38, 39]. The mutation in *SAUSA300_1720* is predicted to be damaging to the protein. This should have a similar effect to a reduced expression of the gene, causing a thickened cell wall. Though promising, we were not able to create a deletion mutation of this gene.

The gene *SAUSA300_1797* is inferred to be a transcriptional regulator and is highly conserved amongst subspecies of *S*. *aureus*. This protein is also referred to as SA1665 I *S*. *aureus* N315 or XdrA which has been shown to bind to the *mecA* promoter in MRSA CHE482. A deletion of the encoding gene has been shown to increase oxacillin resistance in multiple MRSA strains. Although SA1665 has been shown to modulate resistance to β-lactams, an effect on the transcription of *mecA* could not be proven [40]. We have previously shown that exposure of *S*. *aureus* to TDZ reduces the expression of *mecA* significantly after exposure to DCX [4]. This effect could be explained by SA1665 being an inhibitor of *mecA* expression that is activated, when the bacterium is exposed to TDZ. Nevertheless, a deletion mutant of *SAUSA300_1797* did not confirm the gene to be involved in the TDZ resistance mechanism.

The *cls* gene is the second gene that carries a mutation in all TDZ resistant strains. A 13 bp insertion in 50–19 and 60–26 induces a shift in reading frame and thereby a nonfunctional protein. The remaining strains carry a missense mutation, which has been predicted to be damaging to the protein as the residue is a part of a domain with transferase activity. The *cls* gene in *S*. *aureus* USA300 is identical to the *cls2* gene in other, well-studied isolates of *S*. *aureus*. Peleg *et al*. [41] sequenced 21 clinical and 12 laboratory-derived DAP resistant strains and mutations in *cls2* were identified in five of the strains. In two of these strains, *cls2* was the only gene carrying a mutant allele, which resulted in a four-fold increase in the MIC. Another study investigated the transcriptomic effects of DAP resistance in *S*. *aureus* and found that the *cls* gene was significantly downregulated in the laboratory evolved DAP resistant strains [42]. *cls* mutations were also found in DAP resistant *Enterococcus faecium* strains [43], though when the mutated allele was transferred to a DAP-susceptible strain, no change in phenotype was observed [44]. Most papers [41–43] point towards a nonfunctional cardiolipin synthase leading to resistance to DAP, while another paper [45] associates higher cardiolipin content in the cytoplasmic membrane with a reduced susceptibility to DAP. Deletion of the *cls* gene in USA300 results in an increase in the MIC for TDZ to a similar level as the TDZ resistant strain (80–37) indicating a very similar proposed MOA for TDZ and DAP.

### TDZ depolarizes the cytoplasmic membrane

TDZ intercalates in the bacterial membrane through interaction with negatively charged phospholipids, which is quite similar to DAP’s proposed MOA. Therefore, an interesting question to answer was whether TDZ also depolarizes the membrane like DAP [46]. As shown, TDZ lowers the membrane potential drastically, indicating a somewhat similar MOA to DAP. Strains with a TDZ resistant phenotype (80–37 and Δ *cls*) exhibit a similar membrane potential compared to the wild type following TDZ treatment indicating that the membrane potential reducing effect of TDZ is independent of the resistance mechanism.

### Proposed MOA of TDZ

In the search for finding genes directly involved in the TDZ resistance phenotype, generation of individual deletion mutants revealed no genes solely contributing to the phenotype. Only *cls* contributed partly to the phenotype. Hence, several mutations are necessary for the full TDZ resistance phenotype.

TDZ has an effect on the membrane potential, a similar resistance genotype as DAP and both compounds exhibit synergy in combination with β-lactam antibiotics, suggesting that the MOA for these two compounds must be quite similar. To support this, we observed a two-fold increase in resistance for the TDZ resistant strain 80–37 to DAP compared to wild type (data not shown).

Cardiolipin plays a key role in the MOR of TDZ and it is therefore very likely to be an important part of the MOA. TDZ is known to interact with negatively-charged phospholipids, which could indicate that cardiolipin directly interacts with or binds TDZ. Coupling of these two hypotheses leads to the following MOA being proposed: When able to pass through the cell wall, TDZ accumulates in the cytoplasmic membrane through interaction with cardiolipin. Increasing amounts of TDZ compromise the cytoplasmic membrane, leading to ion leakage out of the cell, which lowers and disturbs the electrochemical gradient driving membrane transport of essential molecules. Consequently, this leads to inhibition of life-sustaining processes like the cell wall synthesis, which TDZ is known to affect [4, 5].

## Conclusion

In summary, combining drugs to combat antimicrobial resistance has a great potential in the future but as with all antibiotics, resistance is always an issue. In the present study, we aimed to reveal the mechanism behind TDZ resistance. We found that *S*. *aureus* can become resistant toward TDZ and that cardiolipin seems to play a role in this mechanism. Furthermore, TDZ depolarises the cytoplasmic membrane, a mechanism in which cardiolipin does not seem to be a part of and other genes must be involved.

## Supporting information

S1 Fig Serial passage. Resistance acquisition during serial passaging in the presence of sub-MIC levels of thioridazine. A) The y axis is the highest concentration the cells grew in during passaging. B) MIC values for thioridazine during passaging.
(DOCX)

S2 Fig Filtration of variants with Stampy. Flow chart over filtration of variants identified through reference alignment with Stampy. From the identified 133 variants, 122 were excluded due to presence in wildtype (WT) or filtering parameters, leaving the final number of variants on 11.
(DOCX)

S3 Fig Filtration of variants with cortex_var. Flow chart over filtration of variants identified through de novo assembly with cortex_var. From the 982 raw variants, 970 were excluded due to presence in wildtype (WT) or filtering parameters, leaving the final number of variants on 12.
(DOCX)

S1 Table Bacterial strains used in this study. (DOCX)

S2 Table An overview over the primers used in this study. (DOCX)
