# Supplementary material for: Molecular mechanisms of thioridazine resistance in Staphylococcus aureus
Source: PLoS One. 2018 Aug 8;13(8):e0201767. doi: 10.1371/journal.pone.0201767 (PMC6082566; doi:10.1371/journal.pone.0201767)
Supplement: S1 Fig — Resistance acquisition during serial passaging in the presence of sub-MIC levels of thioridazine. A) The y axis is the highest concentration the cells grew in during passaging. B) MIC values for thioridazine during passaging. (DOCX) [file pone.0201767.s001.docx]

**S1 Fig:** Resistance acquisition during serial passaging in the presence of sub-MIC levels of thioridazine. A) The y axis is the highest concentration the cells grew in during passaging. B) MIC values for thioridazine during passaging.

A)

B)
