# Supplementary material for: Molecular mechanisms of thioridazine resistance in Staphylococcus aureus
Source: PLoS One. 2018 Aug 8;13(8):e0201767. doi: 10.1371/journal.pone.0201767 (PMC6082566; doi:10.1371/journal.pone.0201767)
Supplement: S2 Fig — Flow chart over filtration of variants identified through reference alignment with Stampy. From the identified 133 variants, 122 were excluded due to presence in wildtype (WT) or filtering parameters, leaving the final number of variants on 11. (DOCX) [file pone.0201767.s002.docx]

S2 Fig.

Flow chart over filtration of variants identified through reference alignment with Stampy. From the identified 133 variants, 122 were excluded due to presence in wildtype (WT) or filtering parameters, leaving the final number of variants on 11.
