# Supplementary figures and images for: Molecular mechanisms of thioridazine resistance in Staphylococcus aureus
Source: PLoS One. 2018 Aug 8;13(8):e0201767. doi: 10.1371/journal.pone.0201767 (PMC6082566; doi:10.1371/journal.pone.0201767)

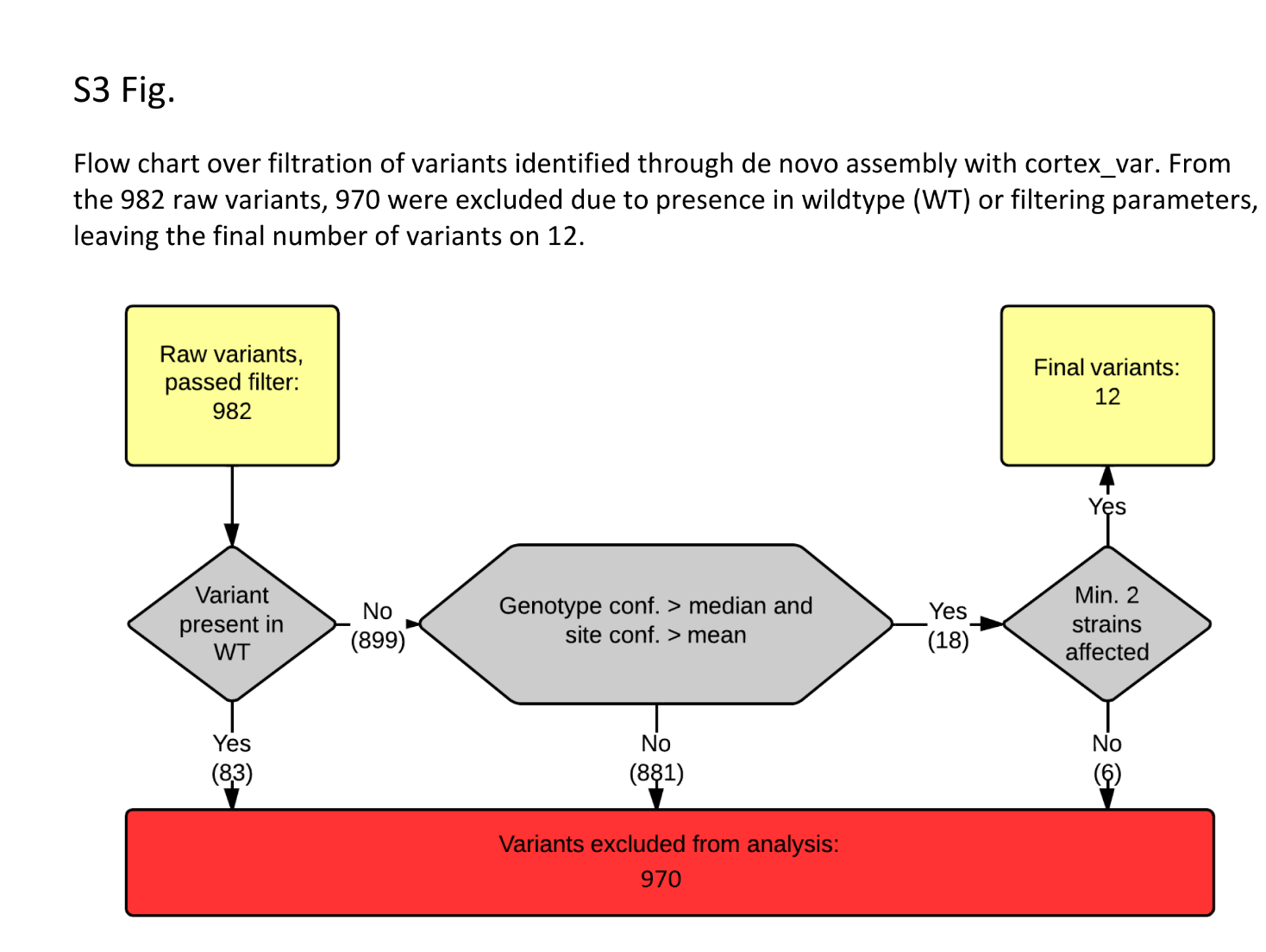

Supplement: S3 Fig — Flow chart over filtration of variants identified through de novo assembly with cortex_var. From the 982 raw variants, 970 were excluded due to presence in wildtype (WT) or filtering parameters, leaving the final number of variants on 12. (DOCX) [file pone.0201767.s003.docx]
