# Supplementary material for: Molecular mechanisms of thioridazine resistance in Staphylococcus aureus
Source: PLoS One. 2018 Aug 8;13(8):e0201767. doi: 10.1371/journal.pone.0201767 (PMC6082566; doi:10.1371/journal.pone.0201767)
Supplement: S1 Table — (DOCX) [file pone.0201767.s004.docx]

S1 Table. Bacterial strains used in this study.

| **Strain** | **Description** | **Source** |
| --- | --- | --- |
| *E. coli* DC10B pIMAY | A sub-strain of *E. coli* DH10B, with knockout of the *dcm* restriction system containing the cloning plasmid pIMAY | [1] |
| *E. coli* IM08B | A sub-strain of DC10B, with insertions of *hsdMS* genes encoding methylase and specificity genes | [2] |
| *S. aureus* USA300 FPR 3757 | Clinical isolate of methicillin-resistant Staphylococcus aureus from a wrist abscess, 36 year old HIV+ male | ATCC nr. BA-1556, <http://www.atcc.org> |
| *S. aureus* USA300 32-3 | *S. aureus* USA300 from a directed evolution experiment where it was grown in increasing concentrations of thioridazine. Isolate from day three, lastly grown in 32 µg/ml of thioridazine. | This work |
| *S. aureus* USA300 40-13 | Isolate from same experiment as *S. aureus* USA300 32-3. From day 13, lastly grown in 40 µg/ml of thioridazine. | This work |
| *S. aureus* USA300 50-19 | Isolate from same experiment as *S. aureus* USA300 32-3. From day 19, lastly grown in 50 µg/ml of thioridazine. | This work |
| *S. aureus* USA300 60-26 | Isolate from same experiment as *S. aureus* USA300 32-3. From day 26, lastly grown in 60 µg/ml of thioridazine. | This work |
| *S. aureus* USA300 70-31 | Isolate from same experiment as *S. aureus* USA300 32-3. From day 31, lastly grown in 70 µg/ml of thioridazine. | This work |
| *S. aureus* USA300 80-37 | Isolate from same experiment as *S. aureus* USA300 32-3. From day 37, lastly grown in 80 µg/ml of thioridazine. | This work |
| *S. aureus* USA300 90-38 | Isolate from same experiment as *S. aureus* USA300 32-3. From day 38, lastly grown in 90 µg/ml of thioridazine. | This work |
| *S. aureus* ΔSAUSA300_0649 | *S. aureus* USA300 with knockout of the gene SAUSA300_0649, which codes for a putative phosphate transport regulator | This work |
| *S. aureus* ΔSAUSA300_0911 | *S. aureus* USA300 with knockout of the gene SAUSA300_0911, which codes for a putative Na+/H+ transporter | This work |
| *S. aureus* USA300_Δ*cls* | *S. aureus* USA300 with knockout of the gene *cls*, which codes for a cardiolipin synthetase | This work |
| *S. aureus* USA300_Δ*pyc* | *S. aureus* USA300 with knockout of the gene *pyc*, which codes for a pyruvate carboxylase | This work |
| *S. aureus* SAUSA300_Δ1797 | *S. aureus* USA300 with knockout of the gene SAUSA300_1797, which codes for a putative transcriptional regulator | This work |
|  |  |  |

1. Monk IR, Shah IM, Xu M, Tan MW, Foster TJ. Transforming the untransformable: application of direct transformation to manipulate genetically *Staphylococcus aureus* and *Staphylococcus epidermidis*. MBio. 2012;3(2). doi: 10.1128/mBio.00277-11. PubMed PMID: 22434850; PubMed Central PMCID: PMC3312211.

2. Monk IR, Tree JJ, Howden BP, Stinear TP, Foster TJ. Complete Bypass of Restriction Systems for Major *Staphylococcus aureus* Lineages. MBio. 2015;6(3):e00308-15. doi: 10.1128/mBio.00308-15. PubMed PMID: 26015493; PubMed Central PMCID: PMCPMC4447248.
