# Supplementary material for: Molecular mechanisms of thioridazine resistance in Staphylococcus aureus
Source: PLoS One. 2018 Aug 8;13(8):e0201767. doi: 10.1371/journal.pone.0201767 (PMC6082566; doi:10.1371/journal.pone.0201767)
Supplement: S2 Table — (DOCX) [file pone.0201767.s005.docx]

S2 Table. An overview over the primers used in this study.

| Name | Description | Sequence | Source |
| --- | --- | --- | --- |
| IM151 | Anneals upstream of the multiple cloning site of the pIMAY plasmid | TACATGTCAAGAATAAACTGCCAAAGC | (1) |
| IM152 | Anneals downstream of the multiple cloning site of the pIMAY plasmid | AATACCTGTGACGGAAGATCACTTCG | (1) |
| rpoC FW | Forward sequencing primer for the *rpoC* gene, used for confirming mutations identified through whole-genome sequencing. | GATCCAGGTCCAACTGGTTTAG |  |
| rpoC RV | Reverse sequencing primer for the *rpoC* gene, used for confirming mutations identified through whole-genome sequencing. | TAAGTTTTGACGGAAACGACCT |  |
| 0649 FW | Forward sequencing primer for the *SAUSA300_0649* gene, used for confirming mutations identified through whole-genome sequencing. | CAATCGCTTAAATGGAGGATTTTA |  |
| 0649 RV | Reverse sequencing primer for the *SAUSA300_0649* gene, used for confirming mutations identified through whole-genome sequencing. | ACATTTATCAGCGATTTCTTCCAT |  |
| 0703 FW | Forward sequencing primer for the *SAUSA300_0703* gene, used for confirming mutations identified through whole-genome sequencing. | GAAACAACGTCAAACAGAGCCTAAC |  |
| 0703 RV | Reverse sequencing primer for the *SAUSA300_0703* gene, used for confirming mutations identified through whole-genome sequencing. | GTTGCATCCTTTTCATCTAATGTG |  |
| 0911 FW | Forward sequencing primer for the *SAUSA300_0911* gene, used for confirming mutations identified through whole-genome sequencing. | TAGCAGGTGTCGTTGTTTCATTAT |  |
| 0911 RV | Reverse sequencing primer for the *SAUSA300_0911* gene, used for confirming mutations identified through whole-genome sequencing. | CAAACAACTATGTCTCGGTCAAAC |  |
| 0911 STOP FW | Forward sequencing primer for the *SAUSA300_0911* gene, used for confirming mutations identified through whole-genome sequencing. | TGTTTCCAGTTCCAGATGAGTTTA |  |
| 0911 STOP RV | Reverse sequencing primer for the *SAUSA300_0911* gene, used for confirming mutations identified through whole-genome sequencing. | TATTACGGATAATACGCACGAAGA |  |
| pyc FW | Forward sequencing primer for the *pyc* gene, used for confirming mutations identified through whole-genome sequencing. | ATTCATCCTGGCTATGGATTTTTA |  |
| pyc RV | Reverse sequencing primer for the *pyc* gene, used for confirming mutations identified through whole-genome sequencing. | ATTCAACAGTACCTGCATTGACAT |  |
| 1119 FW | Forward sequencing primer for the *SAUSA300_1119* gene, used for confirming mutations identified through whole-genome sequencing. | GTAAAAGCCAATGAATCACTTGAAA |  |
| 1119 RV | Reverse sequencing primer for the *SAUSA300_1119* gene, used for confirming mutations identified through whole-genome sequencing. | GGCACCCATTGATTTAAATATCTC |  |
| 1720 FW | Forward sequencing primer for the *SAUSA300_1720* gene, used for confirming mutations identified through whole-genome sequencing. | GCAACTGAAATTAAAAGTGCAATG |  |
| 1720 RV | Reverse sequencing primer for the *SAUSA300_1720* gene, used for confirming mutations identified through whole-genome sequencing. | CGAGTCATAAAAACAACTCGTAGC |  |
| 1797 FW | Forward sequencing primer for the *SAUSA300_1797* gene, used for confirming mutations identified through whole-genome sequencing. | GATAAAATTCCCCAAACAGAAAGGTA |  |
| 1797 RV | Reverse sequencing primer for the *SAUSA300_1797* gene, used for confirming mutations identified through whole-genome sequencing. | CTCTCGAAACAGGTGTGTTACATC |  |
| cls dupl FW | Forward sequencing primer for the *cls* gene, used for confirming mutations identified through whole-genome sequencing. | GACATCTTCTTCAATCATGCGATTA |  |
| cls dupl RV | Reverse sequencing primer for the *cls* gene, used for confirming mutations identified through whole-genome sequencing. | CAGCTAATTGCTCATCAACGATT |  |
| cls FW | Forward sequencing primer for the *cls* gene, used for confirming mutations identified through whole-genome sequencing. | GAAGCATTTTTCCCATCAAAATTAC |  |
| cls RV | Reverse sequencing primer for the *cls* gene, used for confirming mutations identified through whole-genome sequencing. | GTGTAAAAAGCCATTGTCGTAGTG |  |
| 0911 A | Splicing by overlap extension (SOE) primer, FW primer for a 500 BP fragment upstream of the *SAUSA300_0911* gene. Contains a SalI restriction site in 5' end. | GGGGGTCGACCATTTTTAGGAATGATTACTGC |  |
| 0911 B | SOE primer, RV primer for a 500 BP fragment upstream of the *SAUSA300_0911* gene. | CTCCTCCTTAAATTAAGTAAGCC |  |
| 0911 C | SOE primer, FW primer for a 500 BP fragment downstream of the *SAUSA300_0911* gene. Overlaps with primer 0911 B in the 5' end. | GGCTTACTTAATTTAAGGAGGAGCAATAATGATATTAAAATGCAGACG |  |
| 0911 D | SOE primer, RV primer for a 500 BP fragment downstream of the *SAUSA300_0911* gene. Contains a SacI restriction site. | GGGGGAGCTCCAGAAAAGCGTCCGCGTAAG |  |
| 0911 OutF | Anneals upstream of the *SAUSA300_0911* gene, used for confirming loss of gene after allelic exchange. | GATATCGATTCGACTAATGATTC |  |
| 0911 OutR | Anneals downstream of the *SAUSA300_0911* gene, used for confirming loss of gene after allelic exchange. | CTTGAGCTAACAAGAAGCCTTC |  |
| 0649 A | SOE primer, FW primer for a 500 BP fragment upstream of the *SAUSA300 0649* gene. Contains a SalI restriction site in the 5' end. | GGGGGTCGACGTAAATTGTATTAAATGTGAGAAA |  |
| 0649 B | SOE primer, RV primer for a 500 BP fragment upstream of the *SAUSA300 0649* gene. | CTCCATTTAAGCGATTGT |  |
| 0649 C | SOE primer, FW primer for a 500 BP fragment downstream of the *SAUSA300 0649* gene. Overlaps with primer 0649 B in the 5' end. | ACAATCGCTTAAATGGAGGTCATATATAATCATCGTCACTA |  |
| 0649 D | SOE primer, RV primer for a 500 BP fragment downstream of the *SAUSA300 0649* gene. Contains a ClaI restriction site. | GGGGATCGATCTTTAAAAATTGAATACATCAAGAA |  |
| 0649 OutF | Anneals upstream of the *SAUSA300 0649* gene, used for confirming loss of gene after allelic exchange. | GATGCTTTTAATTGAAAAGATTA |  |
| 0649 OutR | Anneals downstream of the *SAUSA300 0649* gene, used for confirming loss of gene after allelic exchange. | GCTCTTGTTAAATTTGCAT |  |
| cls A | SOE primer, FW primer for a 500 BP fragment upstream of the *cls* gene. Contains a SalI restriction site in the 5' end. | GGGGGTCGACCTTGATGAACACCTATCATATTT |  |
| cls B2 | SOE primer, RV primer for a 500 BP fragment upstream of the *cls* gene. | GTTGACGTTGTGTTTTATGG |  |
| cls C2 | SOE primer, FW primer for a 500 BP fragment downstream of the *cls* gene. Overlaps with primer cls B2 in the 5' end. | CCATAAAACACAACGTCAACCAGACGTCATTAGTGCTGC |  |
| cls D2 | SOE primer, RV primer for a 500 BP fragment downstream of the *cls* gene. Contains a SacI restriction site. | GGGGGAGCTCGTTCTGTCCACATAGGTTCAC |  |
| cls OutF | Anneals upstream of the *cls* gene, used for confirming loss of gene after allelic exchange. | GATACAGTTACTTGACCATCTA |  |
| cls OutR | Anneals downstream of the *cls* gene, used for confirming loss of gene after allelic exchange. | GTGGCAACTGTTCAACT |  |
| SAUSA300_0703 A ny | SOE primer, FW primer for a 500 BP fragment upstream of the *SAUSA300_0703* gene. Contains a SalI restriction site in the 5' end. | GGGGGTCGACCGATGCAAATAATTTCCTCCTAATG |  |
| SAUSA300_0703 B | SOE primer, RV primer for a 500 BP fragment upstream of the *SAUSA300_0703* gene. | CGTTTAAAAGTTAACATTGTCATAA |  |
| SAUSA300_0703 C | SOE primer, FW primer for a 500 BP fragment downstream of the *SAUSA300_0703* gene. Overlaps with primer SAUSA300_0703 B in the 5' end. | TTATGACAATGTTAACTTTTAAACGGATAAAATATCAATCCGGGT |  |
| SAUSA300_0703 D | SOE primer, RV primer for a 500 BP fragment downstream of the *SAUSA300_0703* gene. Contains a SacI restriction site. | GGGGGAGCTCCTGTAAAATCATCATCAATACCAC |  |
| SAUSA300_0703 Out F | Anneals upstream of the *SAUSA300_0703* gene, used for confirming loss of gene after allelic exchange. | GCAATTAGAAATGATGAAATAATA |  |
| SAUSA300_0703 Out R | Anneals downstream of the *SAUSA300_0703* gene, used for confirming loss of gene after allelic exchange. | CGATATTGATTTGGATGC |  |
| pyc A | SOE primer, FW primer for a 500 BP fragment upstream of the *pyc* gene. Contains a SalI restriction site in the 5' end. | GGGGGTCGACGATGAGTTCAATAACGGAAGT |  |
| pyc B | SOE primer, RV primer for a 500 BP fragment upstream of the *pyc* gene. | CACTCCTCAAACTATTAGAATTTT |  |
| pyc C | SOE primer, FW primer for a 500 BP fragment downstream of the *pyc* gene. Overlaps with primer pyc B in the 5' end. | AAAATTCTAATAGTTTGAGGAGTGCAATTGACGTGTAATCTCGT |  |
| pyc D | SOE primer, RV primer for a 500 BP fragment downstream of the *pyc* gene. Contains a SacI restriction site. | GGGGGAGCTCCGTATCATGGCTTTTATAGTG |  |
| pyc Out F | Anneals upstream of the *pyc* gene, used for confirming loss of gene after allelic exchange. | GGCTATTTATATACTCTTTGTCAA |  |
| pyc Out R | Anneals downstream of the *pyc* gene, used for confirming loss of gene after allelic exchange. | CTGGTGCACTAGTGAGACAT |  |
| SAUSA300_1119 A | SOE primer, FW primer for a 500 BP fragment upstream of the *SAUSA300_1119* gene. Contains a SalI restriction site in the 5' end. | GGGGGTCGACGCGTTTATGACTACACTAAGTG |  |
| SAUSA300_1119 B | SOE primer, RV primer for a 500 BP fragment upstream of the *SAUSA300_1119* gene. | CTAAGCTTTCTTGCCTGT |  |
| SAUSA300_1119 C | SOE primer, FW primer for a 500 BP fragment downstream of the *SAUSA300_1119* gene. Overlaps with primer SAUSA300_1119 B in the 5' end. | ACAGGCAAGAAAGCTTAGCGTTCTCGAGTTGCTAA |  |
| SAUSA300_1119 D | SOE primer, RV primer for a 500 BP fragment downstream of the *SAUSA300_1119* gene. Contains a SacI restriction site. | GGGGGAGCTCGAGCTGTATATACTTGTCCTTCT |  |
| SAUSA300_1119 Out F | Anneals upstream of the *SAUSA300_1119* gene, used for confirming loss of gene after allelic exchange. | GTTGTACCATTGATATTTTATAAA |  |
| SAUSA300_1119 Out R | Anneals downstream of the *SAUSA300_1119* gene, used for confirming loss of gene after allelic exchange. | CAGCAATATTATTTACCATTAA |  |
| SAUSA300_1720 A | SOE primer, FW primer for a 500 BP fragment upstream of the *SAUSA300_1720* gene. Contains a SalI restriction site in the 5' end. | GGGGGTCGACCCAACAAATGTTAAGAAAGA |  |
| SAUSA300_1720 B | SOE primer, RV primer for a 500 BP fragment upstream of the *SAUSA300_1720* gene. | GAAAACACATTGATTGATAATC |  |
| SAUSA300_1720 C | SOE primer, FW primer for a 500 BP fragment downstream of the *SAUSA300_1720* gene. Overlaps with primer SAUSA300_1720 B in the 5' end. | GATTATCAATCAATGTGTTTTCCATTCAGTTTGACTACGATAAT |  |
| SAUSA300_1720 D | SOE primer, RV primer for a 500 BP fragment downstream of the *SAUSA300_1720* gene. Contains a SacI restriction site. | GGGGGAGCTCCGTGATGATAAATAACTTCACT |  |
| SAUSA300_1720 Out F | Anneals upstream of the *SAUSA300_1*720 gene, used for confirming loss of gene after allelic exchange. | GTAGTGATGCAGACGTAAATTG |  |
| SAUSA300_1720 Out R | Anneals downstream of the *SAUSA300_1720* gene, used for confirming loss of gene after allelic exchange. | GTAACAATGGATTACGCACA |  |
| SAUSA300_1797 A | SOE primer, FW primer for a 500 BP fragment upstream of the *SAUSA300_1797* gene. Contains a SalI restriction site in the 5' end. | GGGGGTCGACGATTCTATTGCTTGCTGTG |  |
| SAUSA300_1797 B | SOE primer, RV primer for a 500 BP fragment upstream of the *SAUSA300_1797* gene. | GTAAACTAATAATAAATTGAGCAGA |  |
| SAUSA300_1797 C | SOE primer, FW primer for a 500 BP fragment downstream of the *SAUSA300_1797* gene. Overlaps with primer SAUSA300_1797 B in the 5' end. | TCTGCTCAATTTATTATTAGTTTACCACATTTCTTGAAACACAGATTA |  |
| SAUSA300_1797 D | SOE primer, RV primer for a 500 BP fragment downstream of the *SAUSA300_1797* gene. Contains a SacI restriction site. | GGGGGAGCTCGTAAAACAAATAAAGAGATTGCA |  |
| SAUSA300_1797 Out F | Anneals upstream of the *SAUSA300_1797* gene, used for confirming loss of gene after allelic exchange. | GAGAAGTAATTTGTTTTATTGACA |  |
| SAUSA300_1797 Out R | Anneals downstream of the *SAUSA300_1797* gene, used for confirming loss of gene after allelic exchange. | CACTACACGAATAAGTTGTCAA |  |
| rpoC A | SOE primer, FW primer for a 500 BP fragment upstream of the *rpoC* gene. Contains a KpnI restriction site in the 5' end. | GGGGGGTACCGCTGCATACACATTACAAGAA |  |
| rpoC B | SOE primer, RV primer for a 500 BP fragment upstream of the *rpoC* gene. | CAATCAAGGAGCCTACCT |  |
| rpoC C | SOE primer, FW primer for a 500 BP fragment downstream of the *rpoC* gene. Overlaps with primer rpoC B in the 5' end. | AGGTAGGCTCCTTGATTGGTTGACGAATTCTCTTGTTC |  |
| rpoC D | SOE primer, RV primer for a 500 BP fragment downstream of the *rpoC* gene. Contains a SacI restriction site. | GGGGGAGCTCGCTACTATTGTCGCATTGACG |  |
| rpoC Out F | Anneals upstream of the *rpoC* gene, used for confirming loss of gene after allelic exchange. | GTTTTGGTGAGATGGAGG |  |
| rpoC Out R | Anneals downstream of the *rpoC* gene, used for confirming loss of gene after allelic exchange. | GCTTTGTCTTGGTTTACGTA |  |
